# Complete mitochondrial genome and phylogenetic analysis of the caddisfly *Neucentropus mandjuricus* (Trichoptera: Polycentropodidae)

**DOI:** 10.1080/23802359.2022.2154622

**Published:** 2022-12-12

**Authors:** Na Ma, Zhen Li, Ting Lei, Chao Yue

**Affiliations:** School of Life Science and Agricultural Engineering, Henan Provincial Key Laboratory of Funiu Mountain Insect Biology, Nanyang Normal University, Nanyang, China

**Keywords:** *Neucentropus*, Polycentropodidae, mitogenome, phylogenetic analysis

## Abstract

We have sequenced and annotated the complete mitogenome of *Neucentropus mandjuricus* Martynov, 1907, a monotypic genus of Polycentropodidae with controversial taxonomic status. The complete mitogenome of *N. mandjuricus* is 15,020 bp in length, including 13 protein-coding genes, two ribosomal genes, 22 transfer RNA genes, and a non-coding control region. The maximum-likelihood and Bayesian’s inference trees based on 13 protein-coding genes of 23 trichopteran species show that *N. mandjuricus* clusters together with the other polycentropodid *Plectrocnemia sp.* with high support value. This study provides a basis for further study on mitogenome and phylogenetics of the Polycentropodidae.

## Introduction

The Polycentropodidae is a large family among Trichoptera and distributed worldwide, consisting of 30 genera in three subfamilies (Morse 2022). *Neucentropus* Martynov, 1907 is a monotypic genus of Polycentropodidae, including only one species: *Neucentropus mandjuricus* Martynov, 1907. However, the taxonomy status of *Neucentropus* has been controversial as the intraspecific variations widely existed in the wing and external genitalia (Li et al. 1998). The phylogenetic analysis based on three protein-coding genes indicated that *Neucentropus* combines with *Neureclipsis* and locates in the most basal branch of Polycentropodidae (Johanson et al. 2012). In this study, we have sequenced and annotated the complete mitogenome of *Neucentropus mandjuricus*, aiming to provide more molecular data for phylogenetic analysis of Polycentropodidae.

## Materials and methods

**Figure 1.**
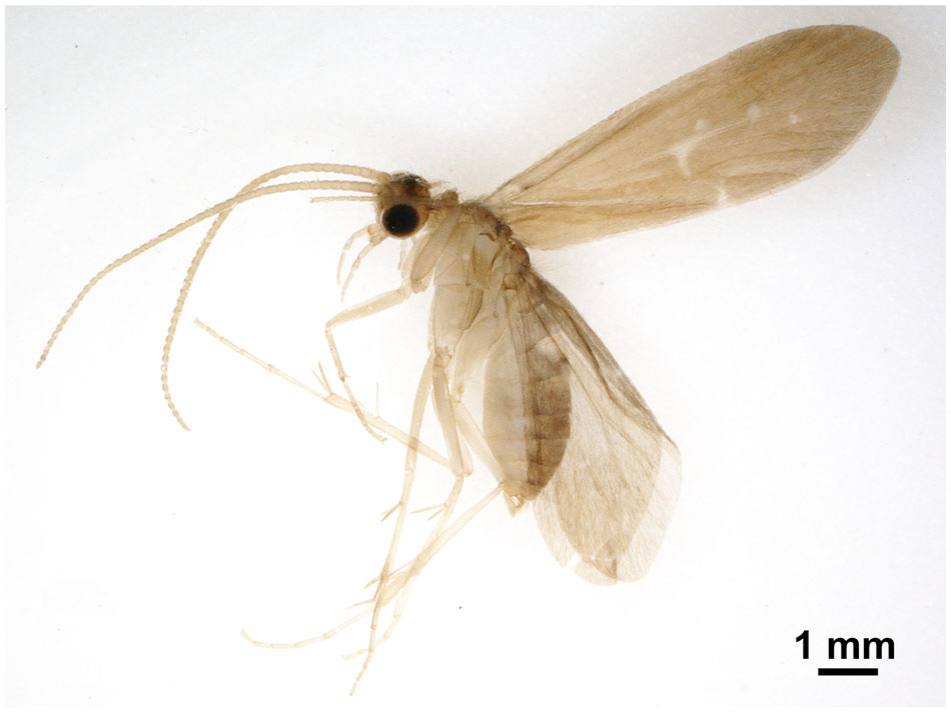
Male adult of *Neucentropus mandjuricus.*

Specimens of *N. mandjuricus* were collected from Xichuan County, Henan Province, China (32°40′25″ N, 111°43′1″ E) in June 2021 (Figure 1), preserved in pure ethanol and transferred to laboratory refrigerator at Nanyang Normal University, Nanyang, China (specimen voucher: NYNU-N1, Chao Yue, yuechaomail@163.com). Whole-genome sequencing of *N. mandjuricus* was performed by Novogene (Beijing, China) using Illumina NovaSeq 6000 platform, the clean reads were subjected to MitoZ v3.3 (Meng et al. 2019) pipeline for mitogenome assembly and annotation. The complete mitogenome of *N. mandjuricus* has been deposited in GenBank under accession number OK509078. The circular mitogenome map was generated using OGDRAW v1.3.1 (Greiner et al. 2019).

## Results and discussion

**Figure 2.**
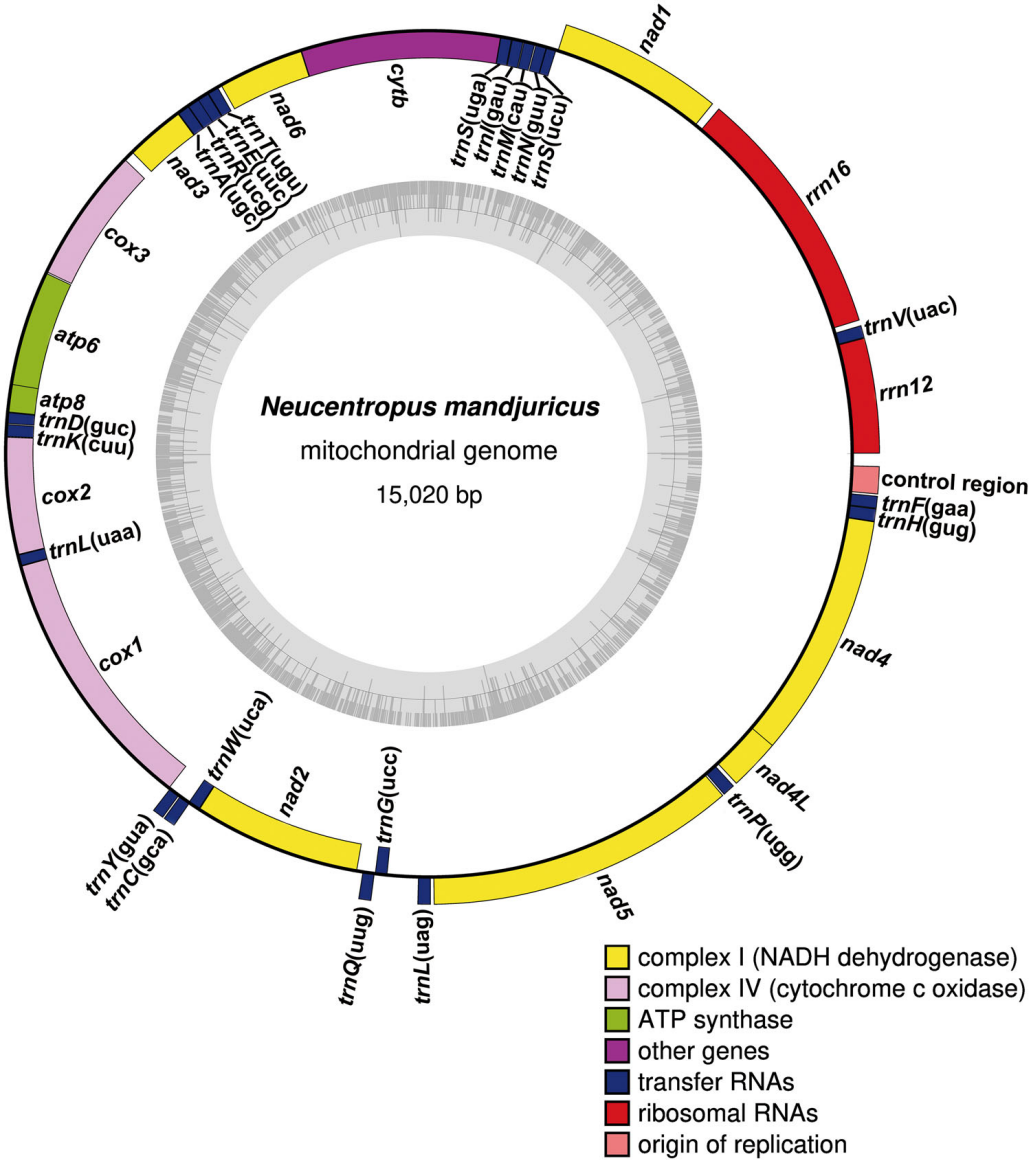
The circular mitogenome map of *Neucentropus mandjuricus.*

The complete mitogenome of *N. mandjuricus* is 15,020 bp in length, with double circular strands consisting of 13 protein-coding genes (PCGs), two ribosomal genes (rRNA), 22 transfer RNA (tRNA) genes, and a non-coding control region (Figure 2). The mitogenome exhibits AT-bias, with the AT content of 81% (A = 37%, T = 44%, C = 6.3%, G = 12.6%). The total length of 13 PCGs is 10,922 bp, accounting for 72.72% of the whole genome. All PCGs are initiated with ATN codons, as four PCGs (*nad3*, *nad4L*, *nad5*, and *nad6*) with ATT, four (*atp6*, *cytb*, *nad1*, and *nad4*) with ATG, four (*nad2*, *cox1*, *cox3*, and *atp8*) with ATA, and *cox2* with ATC. *Nad2*, *cox1*, and *cox2* use the truncated stop codon (T), while the other PCGs all end with TAA. The 22 tRNA genes vary from 58 bp (*trnS*) to 72 bp (*trnK* and *trnL*). Two rRNA genes (*rrn12* and *rrn16*), located at *trnF*/control region and *trnV*/*nad1*, are 622 bp and 1368 bp in length, respectively. Comparative mitogenome analyses between *N. mandjuricus* and *Plectrocnemia sp.* (another Polycentropodinae with complete mitogenome available at GenBank under accession number MW413804) reveals significant collinearity for 13 PCGs. Gene rearrangement is also found for some tRNA genes: *trnG* is located between *trnQ* and *trnL* in *N. mandjuricus*, whereas in *Plectrocnemia sp.* it is between *trnH* and control region.

**Figure 3.**
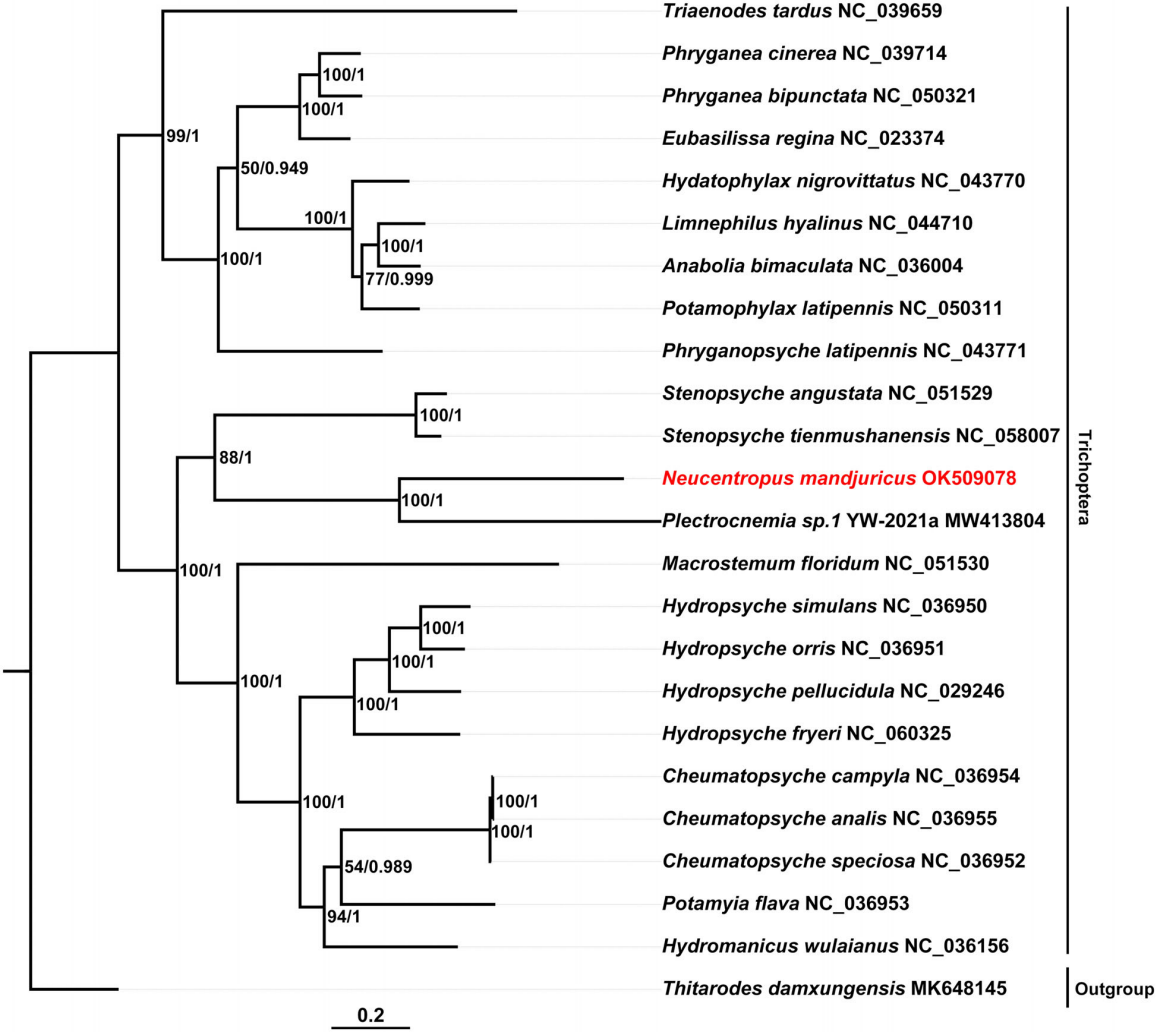
Maximum-likelihood (ML) and Bayesian’s inference (BI) phylogenetic tree generated using concatenated coding sequences of 13 mitochondrial PCGs from 23 trichopteran species, and a Lepidoptera species *Thitarodes damxungensis* as an outgroup. Support values beside nodes represent bootstrap support and posterior probability.

For phylogenetic analysis, the coding sequences of 13 PCGs from 23 Trichoptera, along with a Lepidoptera species *Thitarodes damxungensis* (Yang, 1995) as an outgroup, were individually aligned based on the codons using PAL2NAL v14 (Suyama et al. 2006), and concatenated to form a single dataset. The maximum-likelihood (ML) and Bayesian’s inference (BI) trees were then generated by RAxML-NG v1.1.0 (Kozlov et al. 2019) and MrBayes v3.2.7 (Ronquist et al. 2012), respectively (Figure 3). The result shows that *N. mandjuricus* forms the sister group to *Plectrocnemia sp.* with high support value (BS = 100, PP = 1). Polycentropodidae and Stenopsychidae have close relationship, forming the sister group to other Annulipalpia. Our study provides more molecular data for further research on evolutionary relationships of Polycentropodidae.

## Data Availability

The genome sequence data that support the findings of this study are openly available in GenBank of NCBI at https://www.ncbi.nlm.nih.gov under the accession no. OK509078. The associated BioProject, SRA, and Bio-Sample numbers are PRJNA858192, SRP386152, and SAMN29673587, respectively.
